# Predominant Infection of CD150^+^ Lymphocytes and Dendritic Cells during Measles Virus Infection of Macaques

**DOI:** 10.1371/journal.ppat.0030178

**Published:** 2007-11-16

**Authors:** Rik L de Swart, Martin Ludlow, Lot de Witte, Yusuke Yanagi, Geert van Amerongen, Stephen McQuaid, Selma Yüksel, Teunis B. H Geijtenbeek, W. Paul Duprex, Albert D. M. E Osterhaus

**Affiliations:** 1 Department of Virology, Erasmus MC, University Medical Center, Rotterdam, The Netherlands; 2 School of Biomedical Sciences, Queen's University of Belfast, United Kingdom; 3 Department of Molecular Cell Biology and Immunology, VU University Medical Center, Amsterdam, The Netherlands; 4 Department of Virology, Kyushu University, Fukuoka, Japan; University of Colorado Health Sciences Center, United States of America

## Abstract

Measles virus (MV) is hypothesized to enter the host by infecting epithelial cells of the respiratory tract, followed by viremia mediated by infected monocytes. However, neither of these cell types express signaling lymphocyte activation molecule (CD150), which has been identified as the receptor for wild-type MV. We have infected rhesus and cynomolgus macaques with a recombinant MV strain expressing enhanced green fluorescent protein (EGFP); thus bringing together the optimal animal model for measles and a virus that can be detected with unprecedented sensitivity. Blood samples and broncho-alveolar lavages were collected every 3 d, and necropsies were performed upon euthanasia 9 or 15 d after infection. EGFP production by MV-infected cells was visualized macroscopically, in both living and sacrificed animals, and microscopically by confocal microscopy and FACS analysis. At the peak of viremia, EGFP fluorescence was detected in skin, respiratory and digestive tract, but most intensely in all lymphoid tissues. B- and T-lymphocytes expressing CD150 were the major target cells for MV infection. Highest percentages (up to 30%) of infected lymphocytes were detected in lymphoid tissues, and the virus preferentially targeted cells with a memory phenotype. Unexpectedly, circulating monocytes did not sustain productive MV infection. In peripheral tissues, large numbers of MV-infected CD11c^+^ MHC class-II^+^ myeloid dendritic cells were detected in conjunction with infected T-lymphocytes, suggesting transmission of MV between these cell types. Fluorescent imaging of MV infection in non-human primates demonstrated a crucial role for lymphocytes and dendritic cells in the pathogenesis of measles and measles-associated immunosuppression.

## Author Summary

Measles remains one of the most important causes of childhood mortality in developing countries. The virus is highly infectious and is spread via the respiratory route. According to textbook descriptions, measles virus first infects respiratory epithelial cells, followed by viremia mediated by infected monocytes. However, this order of events is inconsistent with current knowledge about receptor usage by measles virus strains. In this paper we have revisited the pathogenesis of measles by infecting non-human primates with a recombinant measles virus expressing enhanced green fluorescent protein. An important advantage of this system is that infected cells become fluorescent and can be detected with high sensitivity in living animals as well as tissue samples. Strikingly, at the peak of virus replication all lymphoid tissues were strongly fluorescent, and up to 10% of T-lymphocytes and 30% of B-lymphocytes were infected. In peripheral tissues the virus predominantly infected lymphocytes and dendritic cells, although to a lesser extent respiratory epithelial cells were also infected. We hypothesize that measles virus, like human immunodeficiency virus, utilizes dendritic cells as a vehicle to establish infection of the lymphoid system and cause immunosuppression. This study reshapes our basic view of measles pathogenesis.

## Introduction

In spite of significant progress in global measles control programs, each year infections with measles virus (MV) cause almost half a million deaths in developing countries [1,2]. Measles is associated with a profound but transient immunosuppression, and as a result, opportunistic infections may cause pneumonia, gastroenteritis, and otitis media [3,4]. MV is a member of the family *Paramyxoviridae*, genus *Morbillivirus*, which are enveloped viruses with a single-stranded RNA genome of negative polarity. Morbilliviruses are among the most infectious viruses of mammals, and are predominantly transmitted via the respiratory route [3]. Surprisingly, little is known about the specific cells involved in virus transmission and dissemination throughout the body.

Classical textbook descriptions of measles pathogenesis suggest that MV initially infects epithelial cells of the respiratory tract, subsequently spreads to the regional lymph nodes, and is finally disseminated during a viremic phase mediated by infected monocytes [5–7]. However, epithelial cells and unstimulated monocytes do not express signaling lymphocyte activation molecule (SLAM, CD150), the receptor used by wild-type MV [8,9], making this sequence of events unlikely. Moreover, wild-type MV does not readily infect monocytes or epithelial cell lines in vitro [10–12]. CD150 is expressed on subsets of thymocytes, macrophages, dendritic cells (DCs), and activated B- and T-lymphocytes [8,9]; therefore, these cells are the most likely primary target cells for MV infection.

It has been described that wild-type MV strains replicate efficiently in lymphocytes in vivo [13] and in vitro [14], although it has taken until the early 1990s before lymphoid cells replaced Vero cells as the golden standard for isolation of wild-type MV from clinical samples [15]. A decade ago it was shown that human DCs could be infected with wild-type MV [16–18], which was shown to be mediated by CD150 [19]. Infected DCs produced infectious virus [17] and were able to transmit the virus upon in vivo inoculation [20].

Recent studies with an animal morbillivirus, canine distemper virus in ferrets, also showed a viral tropism that was compatible with CD150-expressing cells: the major infected cell populations were T-and B-lymphocytes and thymocytes [21,22]. In this model, infection of macrophages or DCs could not be demonstrated. However, since infection with this ferret-adapted virus causes mainly neurological symptoms and is almost always fatal, it is difficult to extrapolate these data to measles in humans.

Humans are the only natural host for MV, although measles outbreaks have also occurred in captive non-human primates following contact with human patients [23]. The incubation time of measles is approximately 2 wk, which has made it difficult to investigate the early phases of MV infection in humans. Onset of the typical rash coincides with the appearance of virus-specific neutralizing antibodies and T-lymphocytes, which correlate with a rapid decrease in viral load [3]. Although several small laboratory animal models for MV infection have been developed, none of these closely mimic the pathogenesis of measles in humans [24]. Experimental infection of non-human primates, especially macaques, has proven crucial for studying the pathogenesis of MV infection. Both rhesus (Macaca mulatta) and cynomolgus (Macaca fascicularis) macaques are highly susceptible to MV infection [25,26], although clinical signs such as rash and conjunctivitis may be more prominent in rhesus than in cynomolgus macaques [27–29].

A full-length anti-genomic clone (MV-IC323) was previously generated from a Japanese wild-type MV strain, and the resulting recombinant virus was shown to be virulent in macaques [30]. The virus was shown to utilize CD150, but not CD46, as receptor [30,31]. Subsequently, the gene encoding enhanced green fluorescent protein (EGFP) was inserted in this clone within an additional transcription unit upstream of the MV nucleocapsid gene. The resulting recombinant virus, MV-IC323-EGFP, displayed similar in vitro replication characteristics as its parental IC323 strain, and infected cells produced high amounts of EGFP [31]. Morbillivirus genes are transcribed by a start-stop mechanism from a single promoter in the 3′ leader region [4], resulting in the highest number of mRNAs being transcribed from promoter-proximal transcription units. Thus, in MV-IC323-EGFP-infected cells EGFP is the maximal virally expressed protein, and its amount is directly related to the level of virus replication. We therefore reasoned that experimental infection of macaques with MV-IC323-EGFP would provide an ideal opportunity to study measles pathogenesis at the cellular, tissue, and whole organism level.

## Results

### MV-IC323-EGFP Causes Measles in Macaques

**Figure 1:**
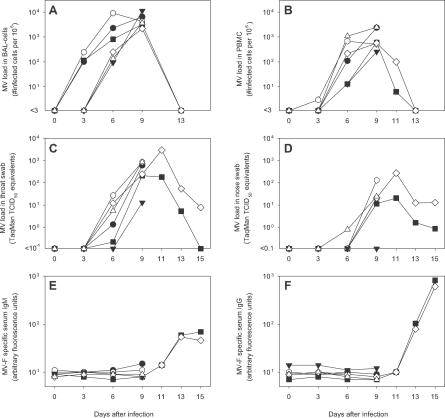
MV Replication and Specific Antibody Responses in Macaques at Different Time Points after Infection Virus isolation from BAL cells (A) and PBMCs (B); virus detection by TaqMan RT-PCR in throat (C) and nose (D) swabs; MV fusion protein (F)–specific serum IgM (E) and IgG (F) responses as determined by FACS-measured immunofluorescence. Symbols indicate rhesus macaques #R1 (•), #R2 (▪), #R3 (▾), and cynomolgus macaques #C1 (○), #C2 (⋄), and #C3 (Δ).

**Figure 2:**
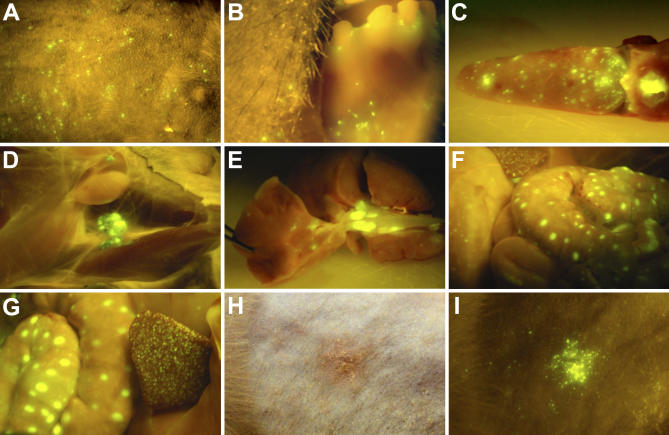
EGFP Fluorescence in Tissues of Macaques after Experimental Infection with MV-IC323-EGFP Cynomolgus macaque #C3 on day 9: MV infection in the skin (A), gingiva and buccal mucosa (B), tongue and tonsils (C), inguinal lymph nodes (D), lungs with tracheo-bronchial lymph nodes (E), stomach (left), spleen (upper left), and large intestine with gut-associated lymphoid tissue (F), spleen (right), and large intestine with gut-associated lymphoid tissue (G); cynomolgus macaque #C2 on day 13 after infection: skin rash shown in normal light (H) or by EGFP fluorescence (I).

Three rhesus (#R1–3) and three cynomolgus macaques (#C1–3) were infected intra-tracheally with 10^4^ cell culture infectious dose-50 of MV-IC323-EGFP. Two animals of each species were euthanized on day 9, the expected time of peak virus replication, and one on day 15, at which time no residual infectious MV was expected to be present. To assess the virulence of the recombinant virus in vivo, we performed virus isolations from broncho-alveolar lavage (BAL) cells and peripheral blood mononuclear cells (PBMCs). MV-IC323-EGFP was isolated from BAL cells and PBMCs of all six animals (Figure 1A and 1B), and kinetics and peak levels of infected cells were similar to those described previously for wild-type MV infection in macaques [25,29,32,33]. MV was also detected by reverse transcriptase (RT)-PCR in throat and nose swabs, but only after onset of viremia (Figure 1C and 1D). MV-specific serum IgM and IgG did not appear until after day 9 (Figure 1E and 1F). Of the two animals that were euthanized at a later time point, one (#C2) developed typical measles skin rash (Figure 2). Collectively, these results indicate that MV-IC323-EGFP infection in macaques is a valid model to study the pathogenesis of measles and is comparable to MV infection in humans.

### Macroscopic Detection of Fluorescence in Skin, Mouth, and Lymphoid Tissues

In the living animals, we examined the skin and mouth for EGFP fluorescence on days 3, 6, and 9 after infection. No fluorescence was detected on day 3, while on day 6 a few fluorescent spots (hallmarks of MV replication) were detected in the buccal mucosa of one animal (#C3). On day 9 after infection, many fluorescent spots were detected in the skin of four of the six animals (Figure 2A): all three cynomolgus macaques and one of the rhesus macaques (#R1). In all six animals fluorescent spots were detected in the buccal mucosa, gingiva, and/or on the tongue on day 9 (Figure 2B and 2C).

Upon necropsy on day 9 we examined the major organs for fluorescence. Lymphoid tissues were brightly fluorescent, including the tonsils, inguinal lymph nodes, tracheo-bronchial lymph nodes, spleen, and gut-associated lymphoid tissue (Figure 2C–2G). Furthermore, fluorescence was also detected in the trachea (unpublished data) and in the wall of the stomach (Figure 2F, left). No fluorescence was visible macroscopically in the thyroid gland, heart, liver, kidneys, adrenal glands, pancreas, urinary bladder, or brain of any of the six animals. Thus, lymphoid tissues were the major sites of MV replication.

On day 11, skin rash was observed on the abdomen and legs of cynomolgus macaque #C2, which co-localized with fluorescence indicating the presence of MV-infected cells in the skin (Figure 2H and 2I). The rash peaked on day 13 and had disappeared on day 15. In rhesus macaque #R2 no fluorescence was detected in the skin at any time point, and this animal did not develop skin rash during the period between day 9 and day 15. The other four animals had been euthanized before the expected time of onset of rash.

Upon necropsy on day 15, fluorescence was no longer macroscopically detectable in lymphoid tissues or any of the other internal organs. However, we still detected fluorescent spots on the skin, tongue, gingiva, and buccal mucosa of one of the two animals (#C2), suggesting delayed clearance in these peripheral tissues. The ability to macroscopically detect MV-infected tissues in the intact host and select these for detailed microscopic analysis provides a unique opportunity for pathogenesis studies.

### Massive MV Replication in B- and T-Lymphocytes

**Figure 3:**
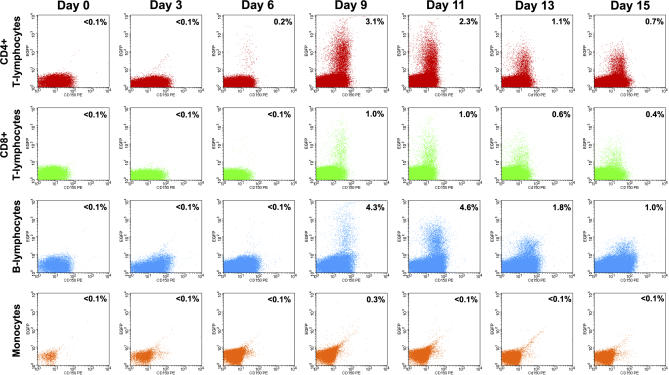
EGFP^+^ Cells in PBMC Subpopulations of Macaque #C2 at Different Time Points after Infection Freshly isolated PBMCs were stained with monoclonal antibodies and analyzed in a FACScalibur measuring approximately 500,000 events per sample to allow detection of low-frequent MV-infected cell populations. Results are shown as dot plots, with EGFP expression on the *y*-axis and CD150 (SLAM) expression on the *x*-axis. EGFP-expression in CD3^+^CD4^+^ T-lymphocytes is shown in red; CD3^+^CD8^+^ T-lymphocytes in green; MHC class-II^+^CD20^+^ B-lymphocytes in blue, and CD14^+^ monocytes in orange.

**Figure 4:**
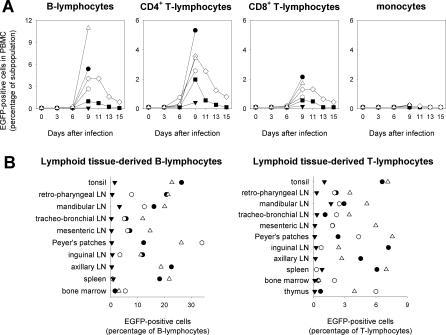
EGFP^+^ Cells in PBMC Subpopulations or in Lymphoid Tissue–Derived B- and T-Lymphocytes Symbols are the same as in Figure 1. Cells were stained with monoclonal antibodies and analyzed in a FACScalibur measuring approximately 500,000 events to allow detection of low-frequency infected cell populations. (A) Percentages of EGFP-positive cells in the same PBMC subpopulations as shown in Figure 3, for all six animals over time. (B) Percentages of EGFP-positive B- and T-lymphocytes in single cell suspensions of lymphoid tissues of the four animals that were euthanized on day 9.

Next, we determined the phenotype of MV-infected cells in peripheral blood and lymphoid tissues by FACS analysis. Between 0.1% and 10% of circulating B-lymphocytes, CD4^+^ T-lymphocytes, and CD8^+^ T-lymphocytes proved to be MV-infected, while MV replication was virtually absent in monocytes (Figures 3 and 4A), natural killer cells, or polymorphonuclear cells (unpublished data). In all PBMC subpopulations, EGFP fluorescence was almost exclusively detected in cells expressing the MV receptor CD150 (Figures 3 and S1). Considering the subpopulation distribution in PBMC (Table S1), the highest absolute numbers of circulating MV-infected cells were detected in the CD4^+^ T-lymphocyte population. Notably, memory T-lymphocytes were preferentially infected, as demonstrated by the two to ten times higher percentage of EGFP^+^ cells in the CD45RA^−^ as compared to the CD45RA^+^ subpopulation of T-lymphocytes (Table S2).

Large numbers of infected lymphocytes were detected in single cell suspensions of lymphoid tissues collected on day 9 after infection, in which up to 10% of T-lymphocytes and up to 30% of B-lymphocytes were EGFP^+^ (Figure 4B). In absolute numbers (Table S3), B-lymphocytes were usually the major infected lymphocyte population in lymphoid tissues. In addition, not only the percentage of infected cells but also the virus replication level, as measured by the level of EGFP expression per cell, was consistently higher in B-lymphocytes than in T-lymphocytes (Figure S2).

### MV Infection in Skin and Mouth

**Figure 5:**
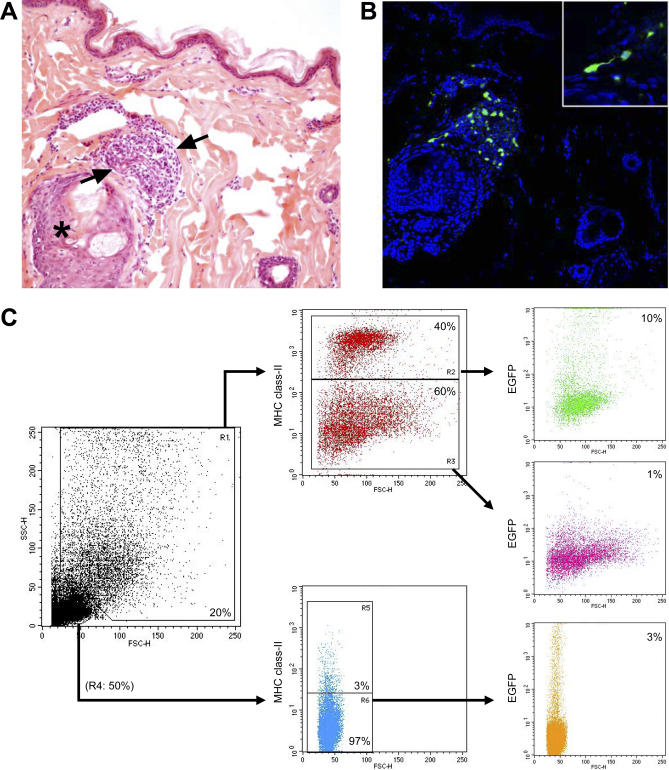
MV-Infected Cells in the Skin of Animal #C3 on Day 9 after Infection (A) and (B) are serial sections of the same tissue. (A) Hematoxylin and eosin staining, showing aggregate of inflammatory cells (between arrows) adjacent to a hair follicle (asterisk). (B) Confocal microscopy image (EGFP-fluorescence in green, TO-PRO counter staining in blue) showing presence of fluorescent cells in the aggregate of inflammatory cells; the inset shows an example of a fluorescent cell with long processes interpreted as a DC. (C) FACS analysis of cells migrated from the dermis after 2 d in cell culture. Half of the migrated cells were small MHC class II-negative (i.e., most likely T-lymphocytes), of which approximately 3% were EGFP-positive. 20% of the migrated cells were identified as large granular cells in scatter, approximately 40% of these were MHC class II-positive (i.e., most likely dendritic cells), of which approximately 10% were EGFP-positive.

In skin samples collected on day 9, we detected MV-infected cells in the dermis but not in the epidermis. Most fluorescent cells were present in association with aggregates of inflammatory cells near hair follicles, and many of the cells had the phenotype of DCs with long cellular processes (Figure 5A and 5B). We placed dermis sections in culture to allow DCs and inflammatory cells to migrate from the tissue, similar as described previously [34]. Fluorescent migrated cells proved to be a mixture of small MHC class-II^−^ and large MHC class-II^+^ cells, suggesting the presence of MV-infected T-lymphocytes and DCs (Figure 5C). In addition, we could isolate MV-IC323-EGFP from these cells in Vero cells expressing CD150.

In the submucosa of tongue and buccal wall, fluorescence was also mainly detected in association with aggregates of inflammatory cells, which in these tissues were present near the mucous glands (Figure S3). Similar to those in the skin, the aggregates appeared to contain both infected lymphocytes and DCs. In addition, in these tissues fluorescent cells were also detected in the keratinized epithelium, in many cases associated with intercellular vacuolization, indicative for epithelial necrosis (Figure S3).

In a biopsy of skin-exhibiting rash that was collected on day 13, we observed sero-purulent crusting. Infected cells were present in aggregates of inflammatory cells close to the hair follicles and sebaceous glands, but a substantial amount of fluorescence was localized to the keratin layer of the stratum corneum (unpublished data).

### MV Infection in Internal Organs

**Figure 6:**
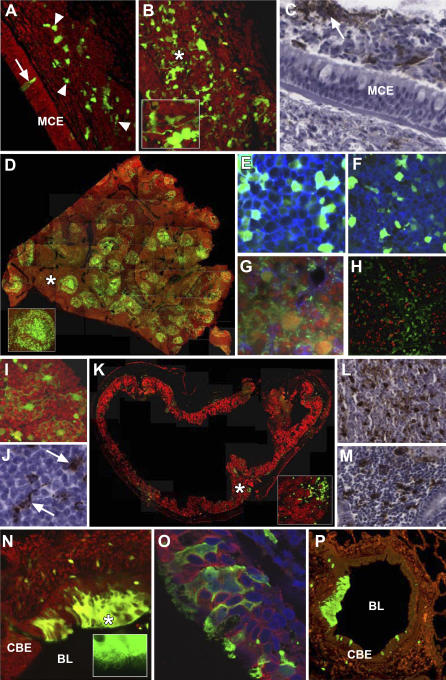
Assessment of the Distribution of MV-Infected Cells in Fixed Tissue Sections Collected 9 d after Infection MV infection was visualized by detection of EGFP fluorescence in paraformaldehyde-fixed vibratome-cut tissue sections (V, 100 μm) or through the use of anti-EGFP or anti-MV N antibodies in formalin-fixed microtome-cut tissue sections (FF, 6 μm) from infected rhesus (#R1) or cynomolgus (#C1) macaques. Propidium iodide (red) was used as a structural counterstain (A, B, D, G, I, N, P). The asterisk indicates the approximate location of the MV-infected cells shown in the inset in (B, D, K and N). (A) Animal #R1 (V). A single MV-infected ciliated epithelial cell (arrow) is visible in the mucociliary epithelium of the tracheal mucosa. A number of MV-infected cells of lymphoid origin (arrowheads) are present in subjacent lamina propria and submucosa. MCE, mucociliary epithelium. (B) Animal #R1 (V). Numerous MV-infected cells (green) with extended cellular processes (inset) are visible in different levels of the tracheal mucosa. (C) Animal #R1 (FF). MV-infected cells (arrow) are present in the tracheal lumen; the arrowhead indicates infected cells in the tracheal lamina propria / submucosa. (D) Animal #R1 (V). A number of MV-infected germinal centers (inset) are visible in a composite image of the spleen. (E) Animal #R1 (V). Co-localization of EGFP fluorescence (green) and CD20-expression (blue) in infected B-lymphocytes in the spleen. (F) Animal #R1 (V). Co-localization of EGFP fluorescence (green) and CD3-expression (blue) in infected T-lymphocytes in the spleen. (G) Animal #R1 (FF). Co-localization of EGFP fluorescence (green) and CD11c-expression (blue) in infected DCs in the spleen. (H) Animal #R1 (FF). No co-localization between MV infection (red) and expression of the macrophage marker Mac287 (green) in the tracheo-bronchial lymph node. (I) Animal #R1 (V). Multiple interconnected foci of MV infection in the tracheo-bronchial lymph node. (J) Animal #R1 (FF). Infection of cells with the morphological characteristics of DCs (arrows) in the tracheo-bronchial lymph node. (K) Animal #R1 (V). Numerous MV-infected cells (green) in a composite image of the ileum. EGFP fluorescence does not co-localize with cytokeratin expression (red) (inset). (L) Animal #R1 (FF). MV-infected cells in lymphoid tissue of the duodenum. (M) Animal #C1 (FF). MV-infected cells in lymphoid tissue of the lamina propria of the stomach. (N) Focus of MV-infected cells (green) in the ciliated bronchial epithelial cell layer of the bronchus adjacent to the bronchial lumen. Fluorescent cilia (inset) are readily identified at the periphery of the infected cells. CBE, ciliated bronchial epithelial cell layer; BL, bronchial lumen. (O) Animal #R1 (FF). MV-infected cells (green) in the bronchus express the epithelial cell marker cytokeratin (red). Cell nuclei were counterstained with DAPI (blue). (P) Animal #C1 (FF). Detection of multiple foci of MV-infected cells (green) in the lung.

Large numbers of MV-infected lymphocytes and DCs were detected in lymphoid tissues and in tissues of the respiratory and digestive tracts collected on day 9 after infection. MV-infected cells in the submucosa of the trachea were interconnected by long dendritic processes (Figure 6A and 6B), suggesting cell-to-cell transmission of the virus. We also detected MV-positive cells in the lumen of the trachea (Figure 6C), which could play a role in virus transmission if expelled during coughing.

Confocal analysis of the spleen showed that red pulp areas contained almost no EGFP^+^ cells, while white pulp areas were strongly fluorescent (Figure 6D). Fluorescent cells were CD20^+^ (Figure 6E), CD3^+^ (Figure 6F), or CD11c^+^ (Figure 6G), but did not co-stain with the macrophage-specific marker Mac287 (Figure 6H, see also Figure S4). Multinucleated EGFP^+^ Warthin-Finkeldey cells (Figure 6I) and EGFP^+^ cells with the distinctive morphology of DCs (Figure 6J) were detected in the lymph nodes.

In the walls of the stomach and the small and large intestines both large process-rich cells (DCs) and small round cells (lymphocytes) were concentrated in aggregates of lymphoid tissue (Figure 6K–6M). EGFP^+^ cells were not detected in the outer epithelial cell layer in these organs. However, EGFP^+^ ciliated epithelial cells were detected in the trachea and in the lungs (Figure 6N–6P). Cell-to-cell fusion of these cells was particularly common in these tissues. Fluorescent ciliated epithelial cells were also detected in BAL samples collected on days 6 and 9 after infection, but the majority of EGFP^+^ cells in the BAL were large MHC class-II^+^ CD11c^+^ cells (most likely alveolar macrophages) or small MHC class-II^−^ CD11c^−^ cells (most likely T-lymphocytes).

## Discussion

Here, we have infected macaques with a recombinant MV strain expressing high levels of EGFP, enabling highly sensitive detection of infected cells. The recombinant virus proved to be virulent in macaques, allowing macroscopic and microscopic assessment of the course of measles. Lymphocytes expressing the MV receptor CD150 were found to be the major target cells for MV replication in vivo. In submucosal tissues we detected large numbers of MV-infected lymphocytes and DCs in aggregates of inflammatory cells. Finally, MV infection was also detected in the epithelia of mouth and trachea.

We identified CD150^+^ B- and T-lymphocytes as major targets for MV infection in vivo. The preferential infection of lymphocytes expressing CD150, a T cell activation marker [35,36], suggests that MV targets activated rather than resting T-lymphocytes. Our in vivo data are in agreement with recently published results of ex vivo infection studies in human tonsillar tissue, demonstrating that wild-type MV strains predominantly infected human CD150^+^ B- and T-lymphocytes [37].

In previous studies monocytes were postulated to be the key MV-infected cell population in peripheral blood of humans, based on the detection of MV RNA by RT-PCR in monocyte-enriched PBMC samples obtained from measles patients [5]. However, the samples used in those studies were collected after onset of rash, and these results may have reflected binding of opsonized virus particles to Fc-receptors rather than active virus replication. Our data are in accordance with the observation that non-activated human monocytes do not express CD150 and are only inefficiently infected in vitro [10,12]. MV-infection of human and non-human primate lymphocytes has been described previously [27,38–40], but the data we collected in macaques are the first to show the kinetics and high frequency of MV-infected lymphocytes in peripheral blood and lymphoid tissues. Estimations of MV-infected cells in PBMCs by an infectious center assay (Figure 1B) or by EGFP detection (Figure 3) suggested that the latter method was one to two log values more sensitive. This clearly illustrates the strength of our model based on infection with an autofluorescent virus as compared to conventional pathogenesis studies.

MHC class II^+^ CD11c^+^ DCs in the dermis of the skin and the submucosa of respiratory and digestive tracts were identified as a second major target cell population for MV infection. Since DCs are known to express CD150 and can be infected with MV in vitro, a role for this cell population in the pathogenesis of measles had been hypothesized [41,42]. Here we formally prove that this is indeed the case in macaques in vivo. Interestingly, infected DCs in our study were in many cases found in association with lymphoid tissue or aggregates of inflammatory cells near hair follicles and mucous glands. Theoretically DCs could also have become fluorescent by uptake of EGFP rather than by MV-infection. However, this is unlikely to result in high EGFP expression levels as those observed in our study (Figures 5 and 6). We are currently undertaking additional studies to further characterize the phenotype and functionality of these infected DCs.

Our observation that high percentages of infected lymphocytes and DCs were detected in lymphoid tissues of infected macaques sheds new light on measles-associated immunosuppression. Measles causes lymphopenia [3], which has previously been attributed to apoptosis of uninfected cells [40]. Our data suggest that actual depletion of the many MV-infected lymphocytes may contribute significantly to the observed lymphopenia. In addition, MV was found to preferentially infect CD150^+^ lymphocytes with a memory phenotype (CD45RA^−^). This is in accordance with data recently published by Condack et al*.*, describing the preferential MV-infection of human memory T-lymphocytes as compared to naive T-lymphocytes in ex vivo infection studies in human tonsillar tissue [37]. Overall, these data strongly suggest that depletion of memory T- and B-lymphocytes as a direct consequence of MV infection may play an important role in immunosuppression. In addition, MV infection of DCs may lead to immune modulation as previously demonstrated in ex vivo studies [41–43].

Levels of MV-infected cells varied substantially between individual animals. Whereas the viral load in PBMCs of animal #R3 resembled those detected in the other animals, the absolute percentages of EGFP-positive cells were much lower (Figure 3B). This animal also had the lowest percentages of EGFP-positive cells in lymphoid tissues (Figure 3C), and no EGFP expression was macroscopically detected in the skin. This variation in responses is likely related to the use of outbred non-SPF animals, and may reflect natural variations in the course of measles in humans, where children may develop either mild or more severe measles.

The case-fatality rate of measles in humans is approximately 0.01% in industrialized countries, but may be up to 10% in developing countries [44]. The increased mortality may be explained by factors such as crowding, poor supportive care, and the high pressure of opportunistic infections [45], which are often considered to be the ultimate cause of measles-associated mortality [3]. However, opportunistic infections may also influence the pathogenesis of measles by another mechanism. Our data demonstrate that CD150^+^ lymphocytes are major targets for MV replication. Opportunistic pathogens may cause chronic immune activation [46], likely resulting in higher numbers of CD150^+^ lymphocytes in circulation and in lymphoid tissues. This suggests that chronic immune activation from high pathogen pressure may facilitate MV replication and spread, thus increasing the severity of MV infection.

Apart from MV infection of lymphocytes and DCs, we also detected MV infection in the squamous stratified epithelium of tongue and buccal mucosa and in the ciliated epithelium of the trachea. In these tissues infection was associated with syncytium formation. Since epithelial cells do not express CD150, these observations suggest the possibility of non-CD150–mediated MV infection and spread. In previous in vitro studies it was shown that MV could infect human primary small airway epithelial cells or lung carcinoma cells via a CD150- and CD46-independent mechanism, resulting in the formation of syncytia [11,47]. It has been suggested that high local concentrations of virus would enable the virus to enter cells using low affinity receptors [9,31]. However, the existence of an additional high affinity MV receptor cannot be excluded [11,47].

On basis of our data we hypothesize that, upon natural MV infection, initial MV replication takes place in the tonsils. However, it remains unclear how the virus gains access to this organ. Given its high person-to-person transmissibility, the virus would need an easily accessible target cell in the upper respiratory or digestive tract that could “trap” the virus and subsequently transport it into lymphoid tissues. The observed substantial MV infection of DCs suggests that CD150^+^ DCs may fulfill this role, as has also been suggested for HIV-1 infection [48]. Notably, DC-SIGN was recently identified as a new attachment receptor for MV, which could enhance viral transmission to CD150^+^ lymphocytes [49]. Whether this process takes place at the respiratory mucosa, in the tonsillar crypts, or elsewhere will need to be determined in future studies focusing on earlier time points after MV infection.

In conclusion, our data demonstrate that in non-human primates MV targets CD150^+^ lymphocytes and CD11c^+^ myeloid cells, and to a lesser extent epithelial cells. In contrast to previous studies in human measles patients, circulating monocytes in peripheral blood do not sustain productive MV infection in macaques. Although the experiments described here are based on a single recombinant MV strain, and important differences may exist between the course of MV infection in humans and macaques, these data warrant re-evaluation of the tropism of MV in humans. This non-human primate model provides new opportunities to address specific aspects of measles pathogenesis in vivo.

## Materials and Methods

### Study design.

Three cynomolgus macaques (animals #C1–3) and three rhesus macaques (animals #R1–3) were infected by intra-tracheal inoculation with 10^4^ cell culture infectious dose-50 of MV-IC323-EGFP diluted to a volume of 5 ml in phosphate buffered saline (PBS). The animals were juvenile (2–4 y), seronegative for measles as determined by virus neutralization, and were housed in negatively pressurized hepa-filtered BSL-3 isolator cages. The virus stock was grown in human B-lymphoblastic B-lymphocytes (BLCL) and tested negative for contamination with *Mycoplasma* species. The infections were performed in pairs, the first (#C1 and #R1) and third (#C3 and #R3) set of animals were euthanized on day 9 while the animals of the second set (#C2 and #R2) were euthanized on day 15. Necropsies were performed in a laminar flow biosafety cabinet. The study was approved by the animal ethics committee and performed according to Dutch guidelines for animal experimentation.

### Samples.

Heparinized blood samples were collected at days 0, 3, 6, 9, 11, 13, and 15 after infection. Plasma was separated by centrifugation, heat inactivated (30 min 56 °C) and stored at −20 °C. PBMCs were isolated by density gradient centrifugation, resuspended in RPMI-1640 supplemented with antibiotics and heat-inactivated fetal bovine serum, counted, and used fresh for virus isolation (see below). BAL were collected on days 3, 6, 9, and 13 after infection, by intra-tracheal infusion of 10-ml PBS through a flexible catheter. Recovered BAL fluid was centrifuged, and BAL-cells were resuspended in culture medium with supplements as described above, counted and used fresh for virus isolation.

### MV detection and serology.

MV was isolated in human BLCL using an infectious centre test as previously described [29,33]. Cytopathic changes were monitored by light and fluorescence microscopy after co-cultivation for 3–6 d, and results were expressed as numbers of infected cells per 10^6^ total cells. In our hands, virus isolation from PBMC and BAL cells in BLCL proved to be slightly more sensitive than isolation in Vero cells expressing CD150 (unpublished data). Real-time RT-PCR was performed as described previously [50]. Fusion protein–specific serum IgM and IgG antibody levels were determined by FACS-measured immunofluorescence as described previously [51].

### Macroscopic detection of EGFP fluorescence.

For the purpose of detecting EGFP fluorescence inside a BSL-3 isolator cage or a laminar flow biosafety cabinet, a lamp was custom-made containing six 5-volt LEDs (Luxeon Lumileds, lambertian, cyan, peak emission 490–495 nm) mounted with D480/40 bandpass filters (Chroma) in a frame that allowed decontamination with 70% alcohol or fumigation with formaldehyde. Emitted fluorescence was visualized through the amber cover of a UV transilluminator (UVP) normally used for screening DNA gels. Photographs were made using a Nikon D80 digital SLR camera.

### Necropsies.

Animals were euthanized by sedation with ketamine (20 mg/kg body weight) followed by exsanguination. Samples were collected both in 4% paraformaldehyde and in buffered formalin. A selection of samples was also collected in PBS for direct processing of fresh tissues or was snap-frozen in liquid nitrogen and stored at −80 °C.

### FACS analysis.

Freshly isolated PBMCs were stained with four different combinations of subset-specific monoclonal antibodies cross-reactive with macaque cells. Staining 1: CD150^PE^ (Pharmingen, clone A12) / CD3^PerCP^ (Pharmingen, clone SP34–2) / CD8^APC^ (DAKO, clone DK25); staining 2: CD150^PE^ / HLA-DR^PerCP^ (BD Biosciences, clone G46–6) / CD20^APC^ (Beckman Coulter, clone B9E9); staining 3: CD150^PE^ / CD14^PerCP^ (Pharmingen, clone M5E2) / CD16^AF647^ (Pharmingen, clone 3G8); staining 4: CD45RA^PE^ (Pharmingen, clone 5H9) / CD3^PerCP^ / CD4^APC^ (BD Biosciences, clone SK3). Fluorescence was detected in a FACSCalibur, obtaining approximately 500,000 events to allow detection of low-frequent EGFP^+^ subpopulations. Lymphoid tissues were minced, and single cell suspensions were prepared by using cell strainers with 100-μm pore size (BD Biosciences); these cells were directly used for FACS analysis.

### Skin cultures.

Shaven skin sections were collected in PBS during necropsies and processed as previously described [34] with adaptations. Briefly, tissues were incubated overnight in trypsin (0.05%), after which dermis and epidermis were mechanically separated. The two tissues were subsequently cultured for 2 d in complete medium to allow DCs to migrate out of the tissue. The single cell suspension that remained in the medium was used for FACS analysis.

### Preparation of tissue samples for vibratome sectioning.

4% (w/v) paraformaldehyde-fixed tissue samples were immersed in 0.2 M sodium cacodylate buffer (pH 7.2) for 20 min and embedded in 5% (w/v) agarose (Type VII low gelling temperature, Sigma) in PBS. A vibratome (Leica Microsystems) was used to cut serial 100-μm sections into 0.2 M TRIS buffered saline. A number of tissue sections were counterstained by incubation for 60 s in propidium iodide. Sections were mounted in Eukitt mounting medium (Electron Microscopy Sciences) onto glass slides and cover slips.

### Immunocytochemical staining of 100-μm vibratome-cut tissue slices.

Vibratome-cut tissue sections (100 μm) were permeabilized in PBS with 0.2% (v/v) Triton X-100 (TX-100) for 30 min at room temperature to facilitate the dissemination of primary and secondary antibodies through the tissue slice. Sections were incubated overnight at 4 °C in an appropriate primary antibody diluted in PBS with 0.1% (v/v) TX-100 and 1% (w/v) bovine serum albumin (BSA). Monoclonal antibodies to CD20 (1:100, DAKO) and CD3 (1:100, DAKO) were used to detect B- and T-lymphocytes, respectively. CAM 5.2 was used to detect cells of an epithelial origin. After incubation in primary antibodies, tissue sections were rinsed three times in PBS with 0.1% (v/v) TX-100. Sections were incubated for 2 h at room temperature in goat anti-mouse Alexa 568 or goat anti-mouse Alexa 647 (Molecular Probes) diluted in PBS with 0.1% TX-100 (v/v) and 1% (w/v) BSA. Sections were rinsed several times in PBS with 0.1% (v/v) TX-100 and mounted as described previously.

### Imaging of vibratome- and microtome-cut brain slices.

Photomicrographs of vibratome-cut tissue slices were collected by confocal scanning laser microscopy. Tissue slices were viewed using an upright DM-IRBE fluorescence microscope (Leica) and appropriate fields selected. A Leica TCS/NT confocal microscope equipped with a krypton-argon laser as the source for the ion beam was used to detect EGFP and appropriate secondary antibodies as described previously [52]. Selected histological or immunocytochemically stained sections were digitally scanned using an Aperio Scanscope T3 with a ×40 objective. From these scans selected images could be viewed or displayed at a range of magnifications.

### Immunohistochemical and immunofluorescence analysis.

All formalin-fixed sections were deparaffinized, and antigen retrieval was performed in a pressure cooker at full power for 2 min in 0.01M TRIS-EDTA buffer (pH 6.0). MV-infected cells were detected using a polyclonal antibody to EGFP (Invitrogen). Sections were incubated in primary antibody overnight at 4 °C, and specific antibody-antigen binding sites were detected using an Envision-Peroxidase system with DAB (DAKO) as substrate. Single and dual labeling immunofluorescence was performed using anti-EGFP and monoclonal antibodies to the DC marker CD11c (Novacastra), the macrophage cell marker Mac387 (Abcam), and the epithelial cell–specific marker CAM 5.2 (Becton-Dickinson). Antigen binding sites were detected with either goat anti-mouse or anti-rabbit Alexa 488 or 568 (Molecular Probes). In some instances, sections were counterstained with either propidium iodide (Sigma) or DAPI mounting medium (Vector).

## Supporting Information

Figure S1 Expression of CD150 on T-Lymphocytes, B-Lymphocytes, and Monocytes PBMCs of animal #R1 collected 9 d after infection with MV-IC323-EGFP were stained with anti-CD150^PE^, anti-CD3^PerCP^, and anti-CD20^APC^. As an isotype control for the anti-CD150 monoclonal, a second sample was stained with mouse IgG1k^PE^, anti-CD3^PerCP^, and anti-CD20^APC^. Three PBMC subpopulations were gated (A): T-lymphocytes (CD3^+^CD20^−^, red), B-lymphocytes (CD3^−^CD20^+^, green), and non-T/non-B cells (blue). The percentages of the total PBMCs and the scatter plots (B) of these subpopulations suggested that the non-T/non-B cells mainly consisted of monocytes. The histograms showed that CD150 was expressed by T-lymphocytes (C) and B-lymphocytes (D), but not by the non-T/non-B cell population (E). Isotype controls are shown as dotted lines.
(2.7 MB TIF)

Figure S2 Percentages of EGFP^+^ Cells in Lymphoid Tissue–Derived Lymphocytes EGFP^+^ lymphocyte subpopulations were detected in single cell suspensions of a mandibular lymph node (A–F) or gut-associated lymphoid tissue (G–L) of macaque #C3, collected 9 d after infection with MV-IC323-EGFP. EGFP-expression in CD3^+^CD8^−^ T-lymphocytes is shown in green (D and J), after gating on region R1 (A and G), and R2 (B and H); EGFP-expression in CD3^+^CD8^+^ T-lymphocytes is shown in purple (E and K), after gating on region R1 (A and G), and R3 (B and H); EGFP-expression in MHC-class II^+^CD20^+^ B-lymphocytes is shown in blue (F and L), after gating on region R1 (A and G), and R4 (C and I).
(2.9 MB TIF)

Figure S3 EGFP^+^ Cells in Tissues of the Oral Cavity Samples were collected from cynomolgus macaque #C3 on day 9 after infection with MV-IC323-EGFP. Subsequent panels represent serial sections of the same tissue, of which the first shows fluorescence (EGFP-fluorescence in green, TO-PRO counter staining in red or blue) and the second the corresponding hematoxylin and eosin staining. EGFP^+^ cells were detected in the lamina propria/submucosa of the tongue (A), seromucous glands of the tongue (C), and buccal wall (E) localized to aggregates of mononuclear cells in these tissues (B, D, and F). Fluorescent cells in the keratinized epithelium of the tongue (G) were detected in association with intercellular vacuolization, indicative for epithelial necrosis (H).
(9.7 MB TIF)

Figure S4 Identification of MV-Infected Cells in Tissue Sections MV-infected cells in paraformaldehyde-fixed vibratome-cut tissue sections (A and B) or formalin-fixed microtome-cut tissue sections (C–E) from macaque #R1 on day 9 after infection with MV-IC323-EGFP.
(A) Identification of MV-infected cells (green) in the spleen that express the B cell marker CD20 (blue).
(B) Identification of MV-infected cells (green) in the spleen that express the T cell marker CD3 (blue).
(C) Identification of MV-infected cells (green) in the spleen that express the DC marker CD11c (blue). Cell nuclei are counterstained with propidium iodide (red).
(D) No co-localization between MV-infected cells (red) in the tracheo-bronchial lymph node and cells expressing the macrophage cell marker Mac287 (green).
(E) MV-infected cells (green) in the bronchus expressing the epithelial cell marker cytokeratin (red). Cell nuclei are counterstained with DAPI (blue).
(2.6 MB TIF)

Table S1 PBMC Lymphocyte Subpopulations Percentages of CD3^+^CD4^+^, CD3^+^CD8^+^, CD20^+^, and CD14^+^ cells in PBMCs collected on different sampling points and percentages of EGFP^+^ cells per PBMC subpopulation.
(64 KB DOC)

Table S2 PBMC T Cell Subpopulations Percentages of CD3^+^CD4^+^CD45RA^−^, CD3^+^CD4^+^CD45RA^+^, CD3^+^CD8^+^CD45RA^−^, and CD3^+^CD8^+^CD45RA^+^ cells in PBMCs collected on different sampling points and percentages of EGFP^+^ cells per subpopulation. In addition, the ratio between the percentage of EGFP^+^ cells in CD45RA^−^ versus CD45RA^+^ cells is shown, indicating preferential MV-infection of CD45RA^−^ T cells (i.e., T cells with a memory phenotype).
(66 KB DOC)

Table S3 Organ Suspension Lymphocyte Subpopulations Percentages of CD3^+^ and CD20^+^ cells in single cell suspensions prepared of different lymphoid tissues collected from animals R1, C1, R3, and C3 and the percentages of EGFP^+^ cells per subpopulation.
(36 KB DOC)

### Accession Numbers

The GenBank (http://www.ncbi.nlm.nih.gov/Genbank/) accession numbers for proteins discussed in this manuscript are CD150 (NP_003028) and EGFP (AAB02574).

## Abbreviations

BAL: broncho-alveolar lavage
DC: dendritic cell
EGFP: enhanced green fluorescent protein
MV: measles virus
PBMC: peripheral blood mononuclear cell
RT-PCR: reverse transcriptase PCR
