# Correction: Predominant Infection of CD150^+^ Lymphocytes and Dendritic Cells during Measles Virus Infection of Macaques

**DOI:** 10.1371/annotation/df340d50-1f94-4d8b-a252-1a82a7fa5cc7

**Published:** 2008-12-11

**Authors:** Rik L de Swart, Martin Ludlow, Lot de Witte, Yusuke Yanagi, Geert van Amerongen, Stephen McQuaid, Selma Yüksel, Teunis B. H Geijtenbeek, W. Paul Duprex, Albert D. M. E Osterhaus

A.D.M.E. Osterhaus wishes to declare, for the avoidance of any misunderstanding on competing interests, that he founded and is chief scientific officer of Viroclinics, a company set up in collaboration with Erasmus University. However, for clarification, no materials or support were received from the company, and no agreements were in place concerning the execution or publication of this work.

