# Supplementary material for: Predominant Infection of CD150+ Lymphocytes and Dendritic Cells during Measles Virus Infection of Macaques
Source: PLoS Pathog. 2007 Nov 16;3(11):e178. doi: 10.1371/journal.ppat.0030178 (PMC2077902; doi:10.1371/journal.ppat.0030178)
Supplement: Supplementary file 1 [file ppat.0030178.st001.doc]

**Supporting Table 1: PBMC Lymphocyte Subpopulations**

| Animal / day | **CD3^+^CD4^+^** | | **CD3^+^CD8^+^** | | **CD20^+^** | | **CD14^+^** | | **CD16^+^** | |
| --- | --- | --- | --- | --- | --- | --- | --- | --- | --- | --- |
|  | **Total^1^** | **%gfp^+2^** | **Total** | **%gfp^+^** | **Total** | **%gfp^+^** | **Total** | **%gfp^+^** | **Total** | **gfp^+^** |
|  |  |  |  |  |  |  |  |  |  |  |
| R1 day 0 | 25% | <0.1 | 16% | <0.1 | 43% | <0.1 | 2% | <0.1 | n.t.% | n.t. |
| **R1 day 3** | 19% | <0.1 | 13% | <0.1 | 46% | <0.1 | 2% | <0.1 | n.t.% | n.t. |
| **R1 day 6** | 20% | 0.2 | 14% | <0.1 | 46% | <0.1 | 1% | <0.1 | n.t.% | n.t. |
| **R1 day 9** | 25% | 5.3 | 28% | 2.2 | 19% | 5.4 | 5% | 0.2 | n.t.% | n.t. |
|  |  |  |  |  |  |  |  |  |  |  |
| **C1 day 0** | 37% | <0.1 | 26% | <0.1 | 10% | <0.1 | 1% | <0.1 | n.t.% | n.t. |
| **C1 day 3** | 40% | <0.1 | 23% | <0.1 | 12% | <0.1 | 2% | <0.1 | n.t.% | n.t. |
| **C1 day 6** | 38% | 0.8 | 24% | 0.2 | 11% | <0.1 | 7% | <0.1 | n.t.% | n.t. |
| **C1 day 9** | 37% | 2.6 | 32% | 0.8 | 4% | 2.7 | 3% | <0.1 | n.t.% | n.t. |
|  |  |  |  |  |  |  |  |  |  |  |
| **R2 day 0** | 19% | <0.1 | 36% | <0.1 | 16% | <0.1 | 3% | <0.1 | 17% | <0.1 |
| **R2 day 3** | 25% | <0.1 | 32% | <0.1 | 16% | <0.1 | 10% | <0.1 | 8% | <0.1 |
| **R2 day 6** | 26% | <0.1 | 34% | <0.1 | 11% | <0.1 | 10% | <0.1 | 10% | <0.1 |
| **R2 day 9** | 25% | 2.0 | 23% | 0.6 | 12% | 1.0 | 14% | <0.1 | 17% | <0.1 |
| **R2 day 11** | 26% | 0.6 | 12% | 0.5 | 15% | 0.8 | 7% | <0.1 | 14% | <0.1 |
| **R2 day 13** | 23% | 0.3 | 15% | 0.1 | 11% | 0.3 | 11% | <0.1 | 15% | <0.1 |
| **R2 day 15** | 27% | 0.1 | 11% | <0.1 | 13% | <0.1 | 3% | <0.1 | 2% | <0.1 |
|  |  |  |  |  |  |  |  |  |  |  |
| **C2 day 0** | 30% | <0.1 | 22% | <0.1 | 16% | <0.1 | 2% | <0.1 | 11% | <0.1 |
| **C2 day 3** | 23% | <0.1 | 23% | <0.1 | 10% | <0.1 | 2% | <0.1 | 23% | <0.1 |
| **C2 day 6** | 23% | 0.2 | 17% | <0.1 | 22% | <0.1 | 10% | <0.1 | 17% | <0.1 |
| **C2 day 9** | 35% | 3.6 | 20% | 1.3 | 6% | 4.1 | 7% | 0.2 | 21% | 0.2 |
| **C2 day 11** | 36% | 2.6 | 21% | 1.3 | 14% | 4.4 | 5% | 0.2 | 16% | <0.1 |
| **C2 day 13** | 32% | 1.3 | 26% | 0.7 | 18% | 1.6 | 5% | <0.1 | 10% | <0.1 |
| **C2 day 15** | 35% | 0.8 | 21% | 0.5 | 15% | 0.9 | 4% | <0.1 | 10% | <0.1 |
|  |  |  |  |  |  |  |  |  |  |  |
| **R3 day 0** | 31% | <0.1 | 21% | <0.1 | 31% | <0.1 | 2% | <0.1 | 5% | <0.1 |
| **R3 day 3** | 30% | <0.1 | 17% | <0.1 | 29% | <0.1 | 4% | <0.1 | 3% | <0.1 |
| **R3 day 6** | 29% | <0.1 | 17% | <0.1 | 43% | <0.1 | 2% | <0.1 | 4% | <0.1 |
| **R3 day 9** | 38% | 0.4 | 18% | 0.2 | 30% | <0.1 | 4% | <0.1 | 3% | <0.1 |
|  |  |  |  |  |  |  |  |  |  |  |
| **C3 day 0** | 37% | <0.1 | 20% | <0.1 | 26% | <0.1 | 2% | <0.1 | 3% | <0.1 |
| **C3 day 3** | 25% | <0.1 | 15% | <0.1 | 19% | <0.1 | 3% | <0.1 | 12% | <0.1 |
| **C3 day 6** | 35% | 0.3 | 15% | 0.1 | 33% | 0.3 | 3% | <0.1 | 6% | <0.1 |
| **C3 day 9** | 50% | 3.4 | 19% | 1.7 | 10% | 11.0 | 5% | 0.3 | 8% | <0.1 |

^1^ subpopulation as percentage of total PBMC (after gating on FSC/SSC for viable cells)

^2^ EGFP-positive cells as percentage of subpopulation
