# Supplementary material for: Predominant Infection of CD150+ Lymphocytes and Dendritic Cells during Measles Virus Infection of Macaques
Source: PLoS Pathog. 2007 Nov 16;3(11):e178. doi: 10.1371/journal.ppat.0030178 (PMC2077902; doi:10.1371/journal.ppat.0030178)
Supplement: Supplementary file 2 [file ppat.0030178.st002.doc]

## Supporting Table 2: PBMC T-cell Subpopulations

| Animal / day | **CD3^+^4^+^45RA^-^** | | **CD3^+^4^+^45RA^+^** | | **Ratio^3^** | **CD3^+^8^+^45RA^-^** | | **CD3^+^8^+^45RA^+^** | | **Ratio** |
| --- | --- | --- | --- | --- | --- | --- | --- | --- | --- | --- |
|  | **Total^1^** | **%gfp^+2^** | **Total** | **%gfp^+^** |  | **Total** | **%gfp^+^** | **Total** | **%gfp^+^** |  |
|  |  |  |  |  |  |  |  |  |  |  |
| R1 day 0 | 12% | <0.1 | 15% | <0.1 | n.a. | 4% | <0.1 | 12% | <0.1 | n.a. |
| **R1 day 3** | 10% | <0.1 | 9% | <0.1 | n.a. | 4% | <0.1 | 9% | <0.1 | n.a. |
| **R1 day 6** | 10% | 0.7 | 11% | 0.2 | 3.9 | 4% | 0.4 | 10% | <0.1 | >4 |
| **R1 day 9** | 11% | 10.7 | 11% | 1.6 | 6.5 | 8% | 5.9 | 20% | 1.2 | 4.9 |
|  |  |  |  |  |  |  |  |  |  |  |
| **C1 day 0** | 19% | <0.1 | 20% | <0.1 | n.a. | 8% | <0.1 | 18% | <0.1 | n.a. |
| **C1 day 3** | 23% | <0.1 | 22% | <0.1 | n.a. | 7% | <0.1 | 16% | <0.1 | n.a. |
| **C1 day 6** | 19% | 1.4 | 19% | 0.2 | 5.9 | 6% | 0.4 | 18% | 0.2 | 2.3 |
| **C1 day 9** | 15% | 5.5 | 23% | 0.8 | 7.2 | 8% | 1.8 | 27% | 0.5 | 3.9 |
|  |  |  |  |  |  |  |  |  |  |  |
| **R2 day 0** | 7% | <0.1 | 13% | <0.1 | n.a. | 3% | <0.1 | 31% | <0.1 | n.a. |
| **R2 day 3** | 7% | <0.1 | 19% | <0.1 | n.a. | 2% | <0.1 | 25% | <0.1 | n.a. |
| **R2 day 6** | 6% | 0.2 | 20% | <0.1 | >2 | 2% | <0.1 | 35% | <0.1 | n.a. |
| **R2 day 9** | 7% | 5.0 | 19% | 1.1 | 4.6 | 2% | 1.5 | 19% | 0.3 | 4.9 |
| **R2 day 11** | 8% | 2.0 | 21% | 0.4 | 4.8 | 3% | 0.8 | 21% | 0.3 | 3.0 |
| **R2 day 13** | 7% | 0.5 | 18% | 0.2 | 2.5 | 5% | <0.1 | 22% | <0.1 | n.a. |
| **R2 day 15** | 11% | 0.2 | 24% | <0.1 | >2 | 3% | <0.1 | 26% | <0.1 | n.a. |
|  |  |  |  |  |  |  |  |  |  |  |
| **C2 day 0** | 6% | <0.1 | 24% | <0.1 | n.a. | 4% | <0.1 | 28% | <0.1 | n.a. |
| **C2 day 3** | 6% | <0.1 | 17% | <0.1 | n.a. | 4% | <0.1 | 22% | <0.1 | n.a. |
| **C2 day 6** | 7% | 0.5 | 17% | <0.1 | >5 | 4% | 0.2 | 17% | <0.1 | >2 |
| **C2 day 9** | 10% | 8.7 | 25% | 1.2 | 7.6 | 3% | 5.6 | 20% | 0.5 | 10.7 |
| **C2 day 11** | 6% | 8.6 | 30% | 1.1 | 7.8 | 2% | 4.9 | 23% | 0.5 | 9.6 |
| **C2 day 13** | 7% | 3.3 | 25% | 0.7 | 5.0 | 6% | 1.1 | 24% | 0.4 | 2.8 |
| **C2 day 15** | 8% | 1.7 | 27% | 0.4 | 4.0 | 3% | 1.1 | 25% | 0.2 | 5.9 |
|  |  |  |  |  |  |  |  |  |  |  |
| **R3 day 0** | 13% | <0.1 | 18% | <0.1 | n.a. | 4% | <0.1 | 19% | <0.1 | n.a. |
| **R3 day 3** | 15% | <0.1 | 15% | <0.1 | n.a. | 3% | <0.1 | 14% | <0.1 | n.a. |
| **R3 day 6** | 14% | <0.1 | 15% | <0.1 | n.a. | 3% | <0.1 | 14% | <0.1 | n.a. |
| **R3 day 9** | 24% | 0.6 | 15% | 0.2 | 3.5 | 3% | 0.2 | 14% | <0.1 | >2 |
|  |  |  |  |  |  |  |  |  |  |  |
| **C3 day 0** | 4% | <0.1 | 33% | <0.1 | n.a. | 1% | <0.1 | 26% | <0.1 | n.a. |
| **C3 day 3** | 4% | <0.1 | 21% | <0.1 | n.a. | 2% | <0.1 | 19% | <0.1 | n.a. |
| **C3 day 6** | 4% | 1.4 | 31% | 0.2 | 6.9 | 2% | 1.1 | 19% | 0.1 | 8.2 |
| **C3 day 9** | 9% | 10.1 | 41% | 1.9 | 5.3 | 2% | 6.8 | 21% | 0.7 | 9.3 |

^1^ subpopulation as percentage of total PBMC (after gating on FSC/SSC for mononuclear cells)

^2^ EGFP-positive cells as percentage of subpopulation

^3^ Ratio of the percentage EGFP-positive cells in the CD45RA^-^ subpopulation (i.e. mainly memory T-lymphocytes) versus the percentage in the CD45RA^+^ subpopulation (i.e. mainly naïve T-lymphocytes)
