# Supplementary material for: Predominant Infection of CD150+ Lymphocytes and Dendritic Cells during Measles Virus Infection of Macaques
Source: PLoS Pathog. 2007 Nov 16;3(11):e178. doi: 10.1371/journal.ppat.0030178 (PMC2077902; doi:10.1371/journal.ppat.0030178)
Supplement: Supplementary file 3 [file ppat.0030178.st003.doc]

## Supporting Table 3: Organ Suspension Lymphocyte Subpopulations

|  | **R1 (day 9)** | | **C1 (day 9)** | | **R3 (day 9)** | | **C3 (day 9)** | |
| --- | --- | --- | --- | --- | --- | --- | --- | --- |
|  | **CD3^+1^** | **CD20^+^** | **CD3^+^** | **CD20^+^** | **CD3^+^** | **CD20^+^** | **CD3^+^** | **CD20^+^** |
|  |  |  |  |  |  |  |  |  |
| Tonsil | 17% | 67% | n.t.^2^ | n.t. | 39% | 52% | 36% | 51% |
| **Retro-pharyngeal LN** | 26% | 49% | 41% | 52% | 58% | 40% | 72% | 26% |
| **Mandibular LN** | 17% | 76% | 20% | 75% | 58% | 38% | 64% | 31% |
| **Tracheo-bronchial LN** | 9% | 79% | 24% | 65% | 55% | 39% | 58% | 34% |
| **Mesenteric LN** | 26% | 60% | 44% | 46% | 69% | 20% | 60% | 31% |
| **Peyer’s patches** | 28% | 65% | 29% | 64% | 78% | 13% | 29% | 62% |
| **Inguinal LN** | 10% | 78% | 50% | 39% | 76% | 17% | 76% | 12% |
| **Axillary LN** | 9% | 66% | n.t. | n.t. | 69% | 25% | 84% | 13% |
| **Spleen** | 10% | 48% | 22% | 19% | 49% | 47% | 41% | 49% |
| **Bone marrow** | 9% | 16% | 24% | 65% | 11% | 4% | 24% | 9% |
| **Thymus** | 20% | 1% | 42% | 1% | 35% | 2% | 67% | 2% |

^1^ subpopulation as percentage of single cell suspension (after gating on FSC/SSC for mononuclear cells)

^2^ n.t. = not tested
